# Prognostic value of the lymphocyte-to-C-reactive protein ratio for mortality in geriatric patients with severe dysphagia requiring artificial nutrition: a retrospective secondary analysis

**DOI:** 10.3389/fmed.2026.1823539

**Published:** 2026-06-09

**Authors:** Chong Wang, Lili Lv, Rongrong Ma

**Affiliations:** Department of Rehabilitation Medicine, The Affiliated Jianhu Hospital of Xinglin College, Nantong University, Yancheng, China

**Keywords:** dysphagia, inflammatory markers, lymphocyte-to-C-reactive protein ratio, mortality, prognosis

## Abstract

**Objective:**

While the lymphocyte-to-C-reactive protein ratio (LCR) is prognostic in critical illness, its role in geriatric patients with severe dysphagia requiring artificial nutrition is unclear. We evaluated its association with mortality in this population.

**Methods:**

Utilizing the Dryad Digital Repository, we conducted a retrospective secondary analysis of a single-center study enrolling 248 patients (January 2014–January 2017). The association between log_2_(LCR) and mortality was evaluated using Cox proportional hazards models, and survival was assessed via Kaplan–Meier curves. Predictive performance across different time points was assessed via time-dependent ROC and landmark analyses. ROC AUC and Boruta feature selection were used to compare LCR with other indices. Subgroup analyses were conducted to confirm robustness.

**Results:**

The secondary analysis comprised 248 patients (151 females; mean age: 83.0 ± 9.3 years). Higher log_2_(LCR) levels were associated with lower mortality (adjusted HR: 0.89, 95% CI: 0.83–0.97). Patients in the highest log_2_(LCR) tertile exhibited a 55% lower risk of death compared to those in the lowest tertile (adjusted HR: 0.45, 95% CI: 0.27–0.76; *P* = 0.003, *P* for trend = 0.003). Median survival times increased across tertiles (214, 359 days, and not reached; *P* < 0.0001). Landmark analyses at 360 days demonstrated a pronounced survival benefit for the highest tertile (HR: 0.15; 95% CI: 0.077–0.286). ROC analyses and Boruta feature selection confirmed the prognostic advantage of LCR, and sensitivity analyses verified the overall robustness of these findings.

**Conclusion:**

In geriatric patients with severe dysphagia requiring artificial nutrition, LCR shows an inverse association with mortality. While its predictive value may weaken over extended follow-up, LCR remains a robust indicator for 1-year mortality. Early LCR assessment could help identify high-risk patients, potentially guiding prompt interventions to improve outcomes.

## Introduction

Dysphagia is a prevalent geriatric syndrome associated with severe complications, including malnutrition, aspiration pneumonia, delirium, and dehydration, which collectively drive escalated healthcare costs and mortality (1, 2). Assessment of this condition typically integrates patient-reported outcomes and/or clinician-conducted evaluations (3, 4). Currently, the reported prevalence of dysphagia among the elderly ranges widely from 15 to 47.4% (5–7), a variability largely attributable to differences in study settings and assessment methodologies. With advancing age, the prevalence of dysphagia increases, thereby elevating the risks of adverse outcomes (8, 9). This trend necessitates not only tailored nutritional strategies (10) but also highlights the need for indicators that can facilitate early assessment of adverse prognoses.

Dysphagia is a complex pathophysiological condition that can be broadly classified into oropharyngeal and esophageal types according to the site of dysfunction and underlying etiology (11). Common causes include neurological disorders such as stroke (12) and Parkinson's disease, structural abnormalities, malignant tumors (13), and age-related degenerative changes in swallowing-related muscles and reflexes. In older adults, physiological aging may impair sensory perception, oral and pharyngeal muscle strength, laryngeal elevation, and swallowing coordination, thereby increasing susceptibility to aspiration and inadequate nutritional intake. These processes may further contribute to chronic inflammation, malnutrition, and immune suppression, creating a vicious cycle that accelerates functional decline and increases mortality risk.

Given the clinical importance of swallowing function, research has increasingly prioritized this field, with some studies investigating prognostic factors among patients with swallowing disorders (14, 15). Consequently, biomarkers reflecting these pathophysiological processes have attracted increasing attention as potential prognostic indicators.

In recent years, the lymphocyte-to-C-reactive protein ratio (LCR) has garnered increasing attention as a novel composite biomarker that effectively reflects both immune-nutritional reserves and inflammatory burden simultaneously. Calculated from routine blood tests, the LCR integrates the host's immune defense and nutritional status (reflected by lymphocyte count) with systemic inflammation intensity (reflected by C-reactive protein). A decreased LCR is generally thought to reflect a vulnerable physiological state, driven by elevated systemic inflammation alongside compromised immunity and malnutrition. Accordingly, this profile has frequently been associated with adverse outcomes in cardiovascular diseases, malignancies, and critical care settings (16–21).

Although the prognostic value of LCR has been demonstrated in other conditions, its role in severe dysphagia remains unclear. Older patients with severe dysphagia constitute a unique population burdened by a complex interplay of multimorbidity, frailty, aspiration risk, and nutritional depletion. However, specific prognostic markers for this vulnerable demographic are scarce. Therefore, this study aimed to evaluate the association between baseline LCR and all-cause mortality in geriatric patients with severe dysphagia requiring artificial nutrition. We hypothesized that a lower LCR is associated with an increased risk of mortality. Ultimately, the integration of LCR as a simple prognostic tool may facilitate early risk stratification and guide individualized management for these patients.

## Methods

### Data sources

We conducted a retrospective secondary analysis using a publicly available dataset obtained from the Dryad Digital Repository (https://doi.org/10.5061/dryad.gg407h1). This dataset was derived from a previously published study in 2019 (14), for which ethical approval had been obtained by the original investigators. The dataset contains de-identified patient-level data and is publicly available under a CC0 Public Domain Dedication, permitting reuse for research purposes in accordance with Dryad's data-sharing policy and terms of use.

The dataset included a well-defined cohort of older patients with severe dysphagia requiring artificial nutritional support and contained the key variables required for the present study, including lymphocyte count, C-reactive protein level, relevant clinical covariates, and survival outcomes. Its single-center design may also have reduced heterogeneity in clinical management, laboratory measurements, and data collection procedures, thereby improving the internal consistency of the data. We also obtained written confirmation from the corresponding author of the original study that the dataset could be used for secondary analysis, and both the original publication and the Dryad dataset were appropriately cited. Because this study used only publicly available and de-identified secondary data, additional institutional review board approval and informed consent were not required for the present analysis.

### Study design and participants

This **secondary analysis** enrolled patients with severe dysphagia who received nutritional support via either percutaneous endoscopic gastrostomy (PEG) or total parenteral nutrition (TPN) at Miyanomori Memorial Hospital (Sapporo, Japan) from January 2014 to January 2017. The diagnosis of severe dysphagia was confirmed by videofluoroscopic examination following a comprehensive assessment by a multidisciplinary team comprising physicians, nurses, and speech-language pathologists. Exclusion criteria included patients with terminal malignancies, those undergoing PEG solely for gastric decompression, and procedures performed prior to January 2014. All procedures were conducted in strict compliance with relevant ethical standards and protocols.

The choice between PEG and TPN was determined through a shared decision-making process involving the clinical team, the patients, and their relatives. We extracted clinical data from medical records, which included demographics and baseline characteristics (age, sex, and body mass index); comorbidities (cerebrovascular diseases, severe dementia, neuromuscular diseases, ischemic heart diseases [IHD], chronic heart failure, chronic lung diseases, chronic liver diseases, chronic kidney diseases, and aspiration pneumonia); survival time; and laboratory parameters [total lymphocyte count [TLC], serum albumin, hemoglobin, and C-reactive protein (CRP)]. Laboratory investigations were conducted within the week preceding the commencement of PEG or TPN. The study endpoint was survival duration measured from the start of the procedure. The LCR was defined as the ratio of pre-operative lymphocyte count (number/mm3) to CRP level (mg/dl) (19, 21, 22). To compare the predictive performance of LCR against other established nutritional-inflammatory markers, we additionally calculated the C-reactive protein-to-albumin ratio [CAR; CRP (mg/L) / albumin (g/dl)] and the prognostic nutritional index {PNI; 10 × albumin (g/dl) + 0.005 × lymphocyte count [(number/mm3)]}.

### Statistical analysis

Categorical variables are summarized as *n* (%), and continuous variables as mean (SD) or median (IQR). Baseline characteristics were compared across log_2_(LCR) tertiles using one-way ANOVA for continuous variables and the chi-square test for categorical variables. Because the data exhibited a skewed distribution, we applied a log_2_ transformation for the analysis. Based on baseline Log_2_(LCR) levels, participants were stratified into three tertiles: T1 (< 9.02), T2 (9.03–11.68), and T3 (>11.69).

Cox proportional hazards models were employed to evaluate the relationship between log_2_(LCR) and mortality outcomes in the dysphagic cohort. Covariates were selected based on at least one of the following criteria: (1) clinical relevance supported; (2) a univariable association with the outcome (*p* < 0.10; Supplementary Table 1); or (3) a >10% change in the primary exposure effect estimate upon entry to the model. Multicollinearity was assessed using the variance inflation factor (VIF); values ≥ 5 indicated multicollinearity. Variables exhibiting multicollinearity were excluded from the final model (Supplementary Table 2). The covariate was complete with the exception of sepsis data for 3 patients (1.2%). To maximize statistical power and minimize bias, missing covariates were imputed using multiple imputation by chained equations with five imputations. Four models were constructed: model 1: unadjusted. model 2: adjusted for sex and age. model 3: adjusted for variables in model 2 plus cerebrovascular disease, severe dementia, chronic kidney disease, congestive heart failure, and ischemic heart disease. model 4: adjusted for variables in model 3 plus percutaneous endoscopic gastrostomy, aspiration pneumonia, sepsis, hemoglobin, and serum albumin. We employed restricted cubic splines (RCS) with 4 knots at the 5th, 35th, 65th, and 95th percentiles to assess relationships. Survival curves were generated using the Kaplan–Meier method, with between-group comparisons by the log-rank test. Subgroup analyses were stratified by age (< 80 vs. ≥80 years), sex, serum albumin (< 3 vs. ≥3 g/dl), and the presence or absence of the following factors: cerebrovascular disease, severe dementia, aspiration pneumonia, ischemic heart disease, chronic kidney disease, sepsis, and PEG, with interactions assessed by *P*-values, and results were presented as forest plots.

The predictive performance of LCR for 1-year mortality was evaluated against its individual components (CRP and TLC) and established immunonutritional indices (albumin, CAR, and PNI). To assess potential incremental prognostic value, each biomarker was independently incorporated into a baseline clinical model. The corresponding areas under the receiver operating characteristic curves (AUCs) were compared using the DeLong test. The Boruta algorithm was applied for feature selection to rank the predictive importance of all evaluated variables.

To verify the robustness of our findings, two specific sensitivity analyses were conducted: (1) Analysis of non-imputed data: we repeated the primary multivariable regression analyses in the original complete-case dataset (excluding missing values). (2) Validation within the 360-day landmark window: recognizing the identified time dependency, we specifically validated prognostic stability within the critical 360-day window. The optimal cutoff value of 11.67 for Log_2_(LCR) was determined using the “surv_cutpoint” algorithm (an outcome-oriented method) in the “survminer” R package. Patients were stratified into high- and low-log_2_(LCR) groups based on this threshold. Subsequent Kaplan-Meier survival curves and comprehensive sensitivity analyses (including crude, multivariable-adjusted, propensity score-adjusted, propensity score-matched [PSM], inverse probability of treatment weighting [IPTW], and pairwise algorithm [PA] models) consistently demonstrated significant prognostic separation, reinforcing the reliability of Log_2_(LCR) as a predictor of mortality within 360 days post-admission.

All analyses were performed using R software (version 4.2.2) and Free Statistics software (version 2.3). A two-sided *P*-value < 0.05 was considered statistically significant.

## Results

### Study participants and baseline characteristics

The secondary analysis included 248 patients (151 females [60.9%]; mean age, 83.0 ± 9.3 years). The detailed patient inclusion and exclusion process is illustrated in Supplementary Figure 1. The median follow-up for the overall cohort was 309.0 (122.8, 623.2) days, compared with 544.0 (240.5, 829.5) for censored subjects. Median overall survival was 474 days (95% CI: 362–699 days), with 134 deaths (54.0%) recorded over a maximum follow-up period of 1,463 days. Baseline characteristics stratified by log_2_(LCR) tertiles are summarized in Table 1. Patients in the highest tertile were generally younger, presenting with higher rates of PEG and cardiovascular disease. These patients also exhibited lower frequencies of severe dementia, IHD, congestive heart failure, and aspiration pneumonia. Laboratory profiles for this subgroup showed higher hemoglobin, serum albumin, and TLC levels, concurrent with lower C-reactive protein concentrations. Survival analysis suggested that the highest tertile was associated with lower mortality and extended survival times.

**Table 1:** Baseline characteristics of the study participants.

Variable	Total (*n* = 248)	Log_2_(LCR), T1 (< 9.02; *n* = 83)	Log_2_(LCR), T2 (9.03–11.68; *n* = 82)	Log_2_(LCR), T3 (>11.69; *n* = 83)	*P-*value
Age (years)	83.0 ± 9.3	84.9 ± 7.2	83.1 ± 8.5	81.1 ± 11.4	0.030
Female, *n* (%)	151 (60.9)	43 (51.8)	51 (62.2)	57 (68.7)	0.080
Body mass index (kg/m^2^)	19.2 ± 3.3	18.9 ± 3.3	19.4 ± 3.7	19.4 ± 3.0	0.553
PEG, *n* (%)	180 (72.6)	53 (63.9)	55 (67.1)	72 (86.7)	0.002
Cerebrovascular disease, *n* (%)	132 (53.2)	35 (42.2)	45 (54.9)	52 (62.7)	0.028
Severe dementia, *n* (%)	100 (40.3)	40 (48.2)	38 (46.3)	22 (26.5)	0.007
Ischemic heart disease, *n* (%)	44 (17.7)	22 (26.5)	11 (13.4)	11 (13.3)	0.038
Congestive heart failure, *n* (%)	103 (41.5)	45 (54.2)	35 (42.7)	23 (27.7)	0.002
Chronic kidney disease, *n* (%)	52 (21.0)	20 (24.1)	20 (24.4)	12 (14.5)	0.203
Aspiration pneumonia, *n* (%)	93 (37.5)	44 (53.0)	31 (37.8)	18 (21.7)	< 0.001
Sepsis, *n* (%)	29 (11.8)	9 (11.1)	15 (18.3)	5 (6.1)	0.052
Neuromuscular disease, *n* (%)	14 (5.6)	5 (6.0)	1 (1.2)	8 (9.6)	0.062
Chronic lung disease, *n* (%)	18 (7.3)	8 (9.6)	7 (8.5)	3 (3.6)	0.282
Chronic liver disease, *n* (%)	15 (6.0)	3 (3.6)	9 (11.0)	3 (3.6)	0.104
Hemoglobin (g/dl)	11.0 ± 2.0	10.3 ± 1.9	10.6 ± 1.8	12.0 ± 1.9	< 0.001
Serum albumin (g/dl)	3.1 ± 0.6	2.8 ± 0.6	3.0 ± 0.5	3.5 ± 0.4	< 0.001
Total lymphocyte count (number/mm3)	1,334.3 ± 708.3	939.1 ± 523.9	1,427.3 ± 775.4	1,637.5 ± 618.7	< 0.001
C-reactive protein (mg/dl)	1.0 (0.3, 3.3)	4.4 (3.1, 6.7)	1.0 (0.6, 1.5)	0.2 (0.1, 0.3)	< 0.001
LCR	1,350.3 (327.6, 4,735.3)	192.2 (113.2, 327.3)	1,350.3 (837.3, 2,080.6)	7,984.2 (4,740.2, 20,053.3)	< 0.001
Log_2_(LCR)	10.4 ± 2.6	7.5 ± 1.0	10.4 ± 0.7	13.3 ± 1.3	< 0.001
Death, *n* (%)	134 (54.0)	58 (69.9)	52 (63.4)	24 (28.9)	< 0.001
Follow-up (days)	309.0 (122.8, 623.2)	173.0 (48.0, 432.0)	316.5 (158.5, 575.2)	453.0 (226.5, 723.5)	< 0.001

Data on sepsis were available for 245 patients (three missing). PEG, percutaneous endoscopic gastrostomy; CRP, C-reactive protein; LCR, lymphocyte-to-CRP ratio. Log_2_(LCR) denotes the base-2 logarithm of LCR.

### Kaplan–Meier curve

**Figure 1:**
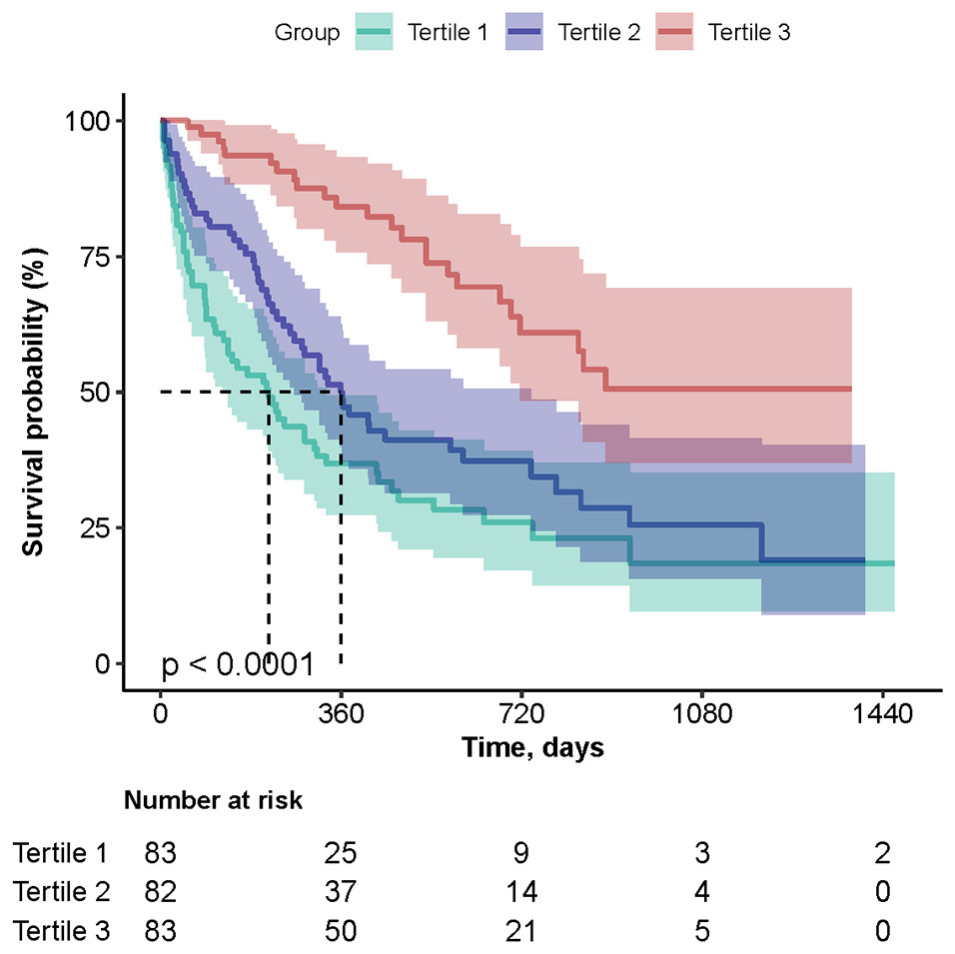
Kaplan–Meier survival curves for mortality stratified by log_2_(LCR) tertiles. Log_2_(LCR) tertiles: T1 (< 9.02), T2 (9.03–11.68), and T3 (>11.69). LCR, lymphocyte-to-C-reactive protein ratio.

The relationship between log_2_(LCR) levels and all-cause mortality was assessed using Kaplan–Meier survival curves. Patients with higher log_2_(LCR) (T3) demonstrated higher cumulative survival rates compared to those with lower levels (T1 and T2; *P* < 0.0001). The median survival time was 214 days for T1 and 359 days for T2, whereas it was not reached for T3 (Figure 1).

### Association between log_2_(LCR) and mortality

Table 2 shows the multivariable Cox proportional hazards regression analysis of log_2_(LCR) and mortality. In the fully adjusted model, log_2_(LCR) treated as a continuous variable was associated with a reduced mortality risk (HR: 0.89 [95% CI: 0.83–0.97], *P* = 0.005). When categorized into tertiles, using T1 (< 9.02) as the reference, the HRs were 0.80 (95% CI: 0.53–1.19; *P* = 0.273) for T2 and 0.45 (95% CI: 0.27–0.76; *P* = 0.003) for T3 (*P* for trend = 0.003). A linear association between log_2_(LCR) and the risk of mortality was identified through RCS analysis (overall *P* = 0.042; non-linearity *P* = 0.808; Supplementary Figure 2).

**Table 2:** Multivariable Cox proportional hazards regression of log_2_(LCR) and mortality.

Variable	Model 1		Model 2		Model 3		Model 4	
	HR (95% CI)	*P-*value	HR (95% CI)	*P-*value	HR (95% CI)	*P-*value	HR (95% CI)	*P-*value
Log_2_(LCR)	0.82 (0.76–0.87)	< 0.001	0.84 (0.78–0.90)	< 0.001	0.85 (0.79–0.92)	< 0.001	0.89 (0.83–0.97)	0.005
Tertile of log_2_(LCR)								
T1 (< 9.02)	1.00 (Ref.)		1.00 (Ref.)		1.00 (Ref.)		1.00 (Ref.)	
T2 (9.03–11.68)	0.69 (0.47–1.00)	< 0.001	0.74 (0.51–1.08)	0.122	0.78 (0.53–1.14)	0.194	0.80 (0.53–1.19)	0.273
T3 (>11.69)	0.25 (0.16–0.41)	< 0.001	0.30 (0.19–0.49)	< 0.001	0.33 (0.21–0.54)	< 0.001	0.45 (0.27–0.76)	0.003
*P* for trend		< 0.001		< 0.001		< 0.001		0.003

Model 1: unadjusted. Model 2: adjusted for sex and age. Model 3: adjusted for variables in model 2 plus cerebrovascular disease, severe dementia, chronic kidney disease, congestive heart failure, and ischemic heart disease. Model 4: adjusted for variables in model 3 plus percutaneous endoscopic gastrostomy, aspiration pneumonia, sepsis, hemoglobin, and serum albumin. HR, hazard ratio; CI, confidence interval; CRP, C-reactive protein; LCR, lymphocyte-to-CRP ratio.

### Subgroup analyses

**Figure 2:**
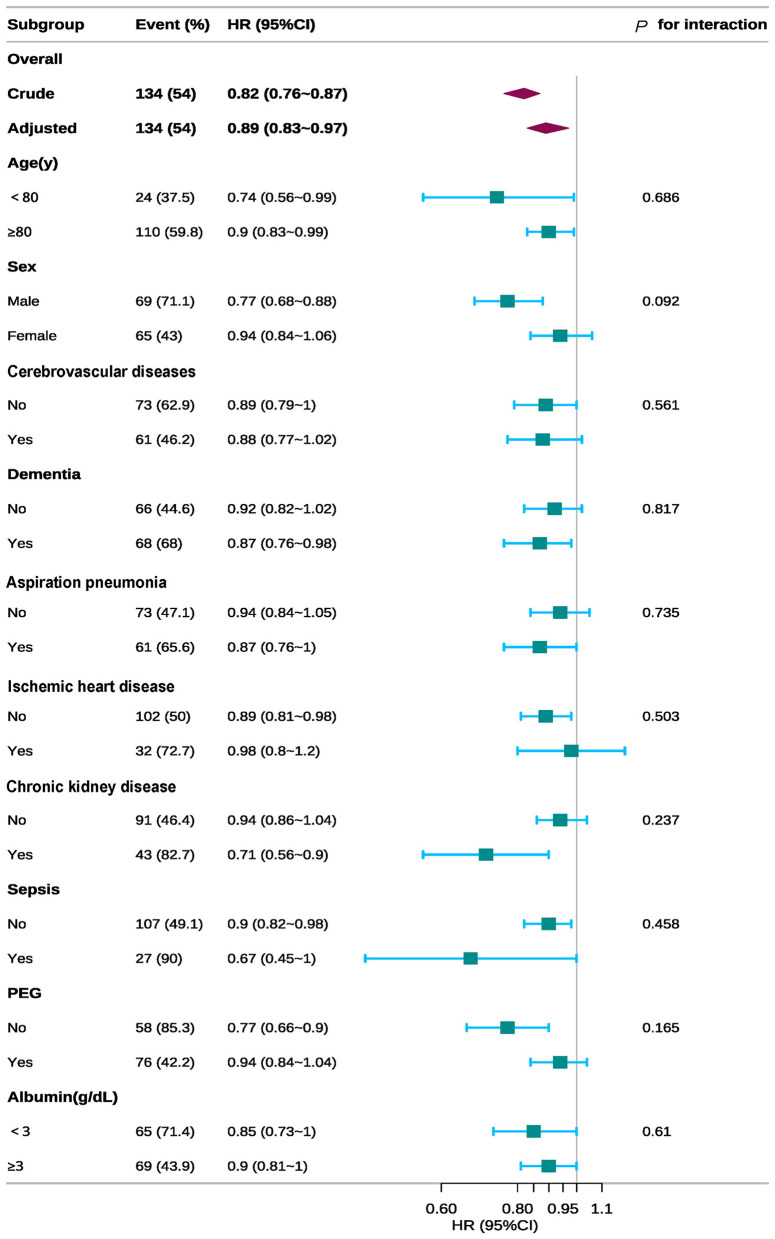
Subgroup analyses of the association between log_2_(LCR) and mortality. Hazard ratios (HRs) were adjusted for age, sex, cerebrovascular disease, severe dementia, chronic kidney disease, congestive heart failure, ischemic heart disease, percutaneous endoscopic gastrostomy, aspiration pneumonia, sepsis, hemoglobin, and serum albumin. LCR, lymphocyte-to-C-reactive protein ratio.

Subgroup analyses were performed to assess the consistency of the association between log_2_(LCR) and mortality. Within the fully adjusted model, this inverse association was generally maintained across all stratified groups, with no significant interactions observed among the subgroups (all *P* for interaction >0.05; Figure 2).

### Temporal dynamics of log_2_(LCR) predictive value

**Figure 3:**
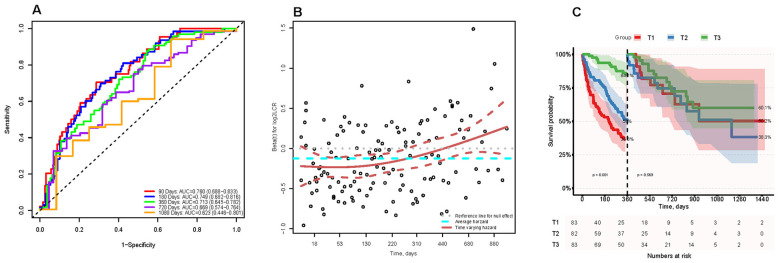
Temporal dynamics of log_2_(LCR) predictive value. **(A)** Time-dependent receiver operating characteristic (ROC) curves for log_2_(LCR) in predicting mortality. AUCs (95% CI) are shown for 90, 180, 360, 720, and 1,080 days. **(B)** Scaled Schoenfeld residual plot for log_2_(LCR). The solid red line shows the time-varying coefficient β(t) {β(t) = ln[HR(t)]} with 95% CIs (red dashed lines), whereas the cyan line represents the average effect. **(C)** Landmark analyses stratified by log_2_(LCR) tertiles. Panels display survival trends within 360 days (left panel, *P* < 0.001) and beyond 360 days (right panel, *P* = 0.569). LCR, lymphocyte-to-C-reactive protein ratio; AUC, area under the curve; CI, confidence interval; ROC, receiver operating characteristic.

Time-dependent ROC analyses demonstrated that log_2_(LCR) maintained robust predictive performance during the early follow-up period, with AUCs of 0.760 (95% CI: 0.688–0.833) at 90 days, 0.749 (0.682–0.816) at 180 days, and 0.713 (0.645–0.782) at 360 days. This discrimination gradually attenuated over time, with AUCs declining to 0.669 (0.574–0.764) at 720 days and 0.623 (0.446–0.801) at 1,080 days. Given the natural disease trajectory in older patients with dysphagia and Schoenfeld residual plots suggesting a time-varying effect, a landmark analysis (360-day cutoff) further evaluated these temporal patterns. During the initial 360 days, individuals in the highest tertile (T3) exhibited a lower mortality hazard compared to those in the lowest tertile (T1; HR: 0.15, 95% CI: 0.077–0.286; *P* < 0.001). Among patients surviving beyond the first year, the prognostic association of baseline log_2_(LCR) diminished and was no longer statistically significant (Figure 3, Table 3).

**Table 3:** Landmark analyses of the association between log_2_(LCR) and mortality.

Variable	No. total	No. events (%)	HR (95% CI)	*P-*value
Time<360 days				
T1 (< 9.02)	83	50 (60.2)	1.00 (Ref.)	
T2 (9.03–11.68)	82	39 (47.6)	0.62 (0.41–0.94)	0.025
T3 (>11.69)	83	11 (13.3)	0.15 (0.08–0.29)	< 0.001
*P* for trend	248	100 (40.3)		< 0.001
Time ≥360 days				
T1 (< 9.02)	25	8 (32.0)	1.00 (Ref.)	
T2 (9.03–11.68)	37	13 (35.1)	1.11 (0.46–2.68)	0.815
T3 (>11.69)	50	13 (26.0)	0.74 (0.31–1.79)	0.508
*P* for trend	112	34 (30.4)		0.424

Log_2_(LCR) tertiles: T1, T2, and T3. HR, hazard ratio; CI, confidence interval; CRP, C-reactive protein; LCR, lymphocyte-to-CRP ratio.

### ROC analyses and Boruta feature selection for 1-year mortality

**Figure 4:**
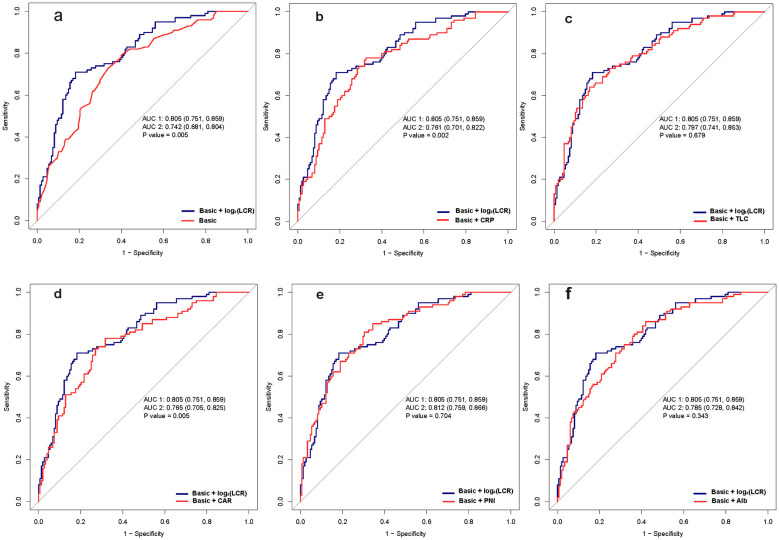
Comparison of ROC curves for 1-year mortality. Basic model adjusted for age and sex. The ROC curves compare the model with basic + log_2_(LCR) (blue line) against the following reference models (red lines): **(a)** the basic clinical model alone, and the basic clinical model combined with **(b)** CRP, **(c)** TLC, **(d)** CAR, **(e)** PNI, and **(f)** Alb.

**Figure 5:**
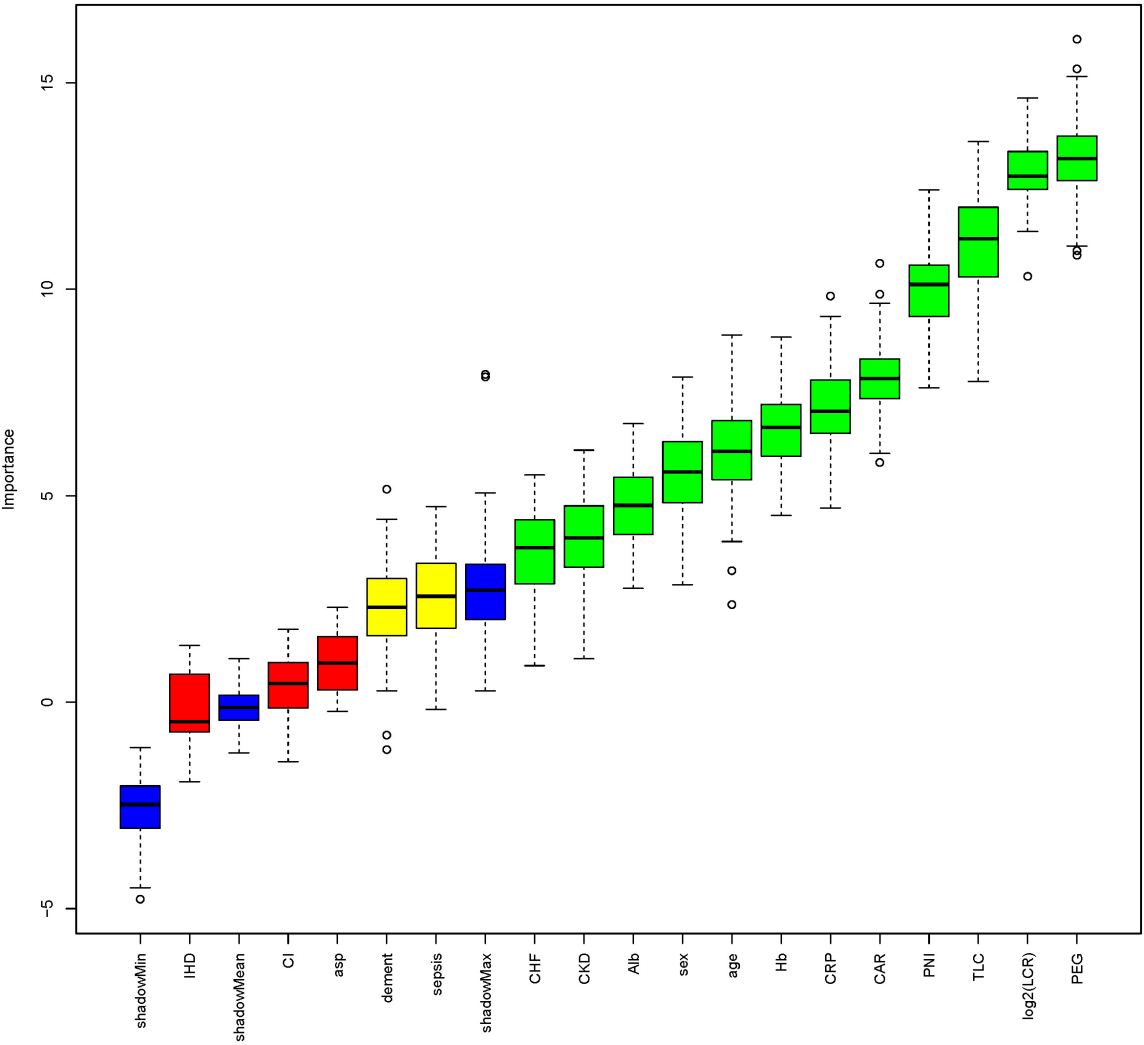
Feature importance ranking for 1-year mortality prediction using the Boruta algorithm. Candidate features are classified as confirmed important (green boxes), tentative (yellow boxes), and rejected (red boxes). Variables are ordered from left to right based on their increasing importance scores; LCR, lymphocyte-to-C-reactive protein ratio; PEG, percutaneous endoscopic gastrostomy; TLC, total lymphocyte count; PNI, prognostic nutritional index; CAR, C-reactive protein-to-albumin ratio; CRP, C-reactive protein; Hb, hemoglobin; Alb, albumin; CKD, chronic kidney disease; CHF, congestive heart failure; Asp, aspiration pneumonia; CI, Cerebrovascular disease; IHD, ischemic heart disease.

ROC analyses indicated that Incorporating log_2_(LCR) into the basic clinical model (age and sex) improved the discrimination for 1-year mortality, increasing the AUC from 0.742 (95% CI: 0.681–0.804) to 0.805 (95% CI: 0.751–0.859; *P* = 0.005; Figure 4a). For 1-year mortality prediction, when evaluating individual markers added to the basic model, log_2_(LCR) outperformed both CRP (AUC: 0.805 [95% CI: 0.751–0.859] vs. 0.761 [95% CI: 0.701–0.822]; *P* = 0.002) and CAR (0.805 [95% CI: 0.751–0.859] vs. 0.765 [95% CI: 0.705–0.825]; *P* = 0.005). Although the addition of PNI yielded a nominally higher AUC [0.812 (95% CI: 0.759–0.866)], DeLong testing indicated this difference was not statistically significant compared with the log_2_(LCR) model (*P* = 0.704). Similarly, no significant differences were observed when incorporating TLC or albumin (all *P* > 0.05) (Figures 4b–f). However, in the Boruta feature selection analysis, although PEG was identified as the most important feature overall, log_2_(LCR) achieved the highest importance score (12.82) among the evaluated inflammatory and nutritional indices, outranking TLC (11.13) and PNI (10.02; Figure 5, Supplementary Table 3). These findings suggest that log_2_(LCR) may provide a potential relative advantage in 1-year prognostic assessment compared with other traditional blood markers for 1-year mortality prediction.

### Sensitivity analyses

To verify the robustness of our findings, we conducted two additional sensitivity analyses: (1) Analysis of non-imputed data: we repeated the primary multivariable analyses using the original complete dataset (excluding missing values). The results remained consistent with the primary findings (Supplementary Table 4). (2) Validation within the 360-day landmark window: to evaluate prognostic stability within the identified critical timeframe, patients were stratified into high- and low-log_2_(LCR) groups using a calculated cutoff of 11.67 (derived from maximally selected rank statistics). Subsequent Kaplan-Meier survival curves and sensitivity analyses indicated significant prognostic separation, supporting the potential utility of log_2_(LCR) as a prognostic indicator within 360 days post-admission (Supplementary Figures 3, 4).

## Discussion

Our study suggests that LCR is a simple prognostic indicator for survival in geriatric patients with severe dysphagia requiring artificial nutrition. Multivariable regression analyses showed that in the fully adjusted model, each 1-unit increase in log_2_(LCR) was associated with an 11% reduction in mortality risk. When stratified by log_2_(LCR) tertiles, patients in the highest tertile (T3) demonstrated a 55% reduction in mortality risk relative to the lowest tertile (T1). This inverse association remained consistent across all evaluated subgroups, with no significant interaction effects observed. Time-dependent analyses and Schoenfeld residual plots showed that the prognostic utility of LCR was primarily evident for 1-year mortality, attenuating to non-significance during extended follow-up. ROC curve comparisons and Boruta feature selection supported the incremental prognostic value and clinical applicability of LCR. Sensitivity analyses maintained the stability of these observations, underscoring LCR's potential to aid in prognostic stratification for this population.

Dysphagia arises from diverse etiologies, including stroke (23), Parkinson's disease (24, 25), dementia (26), cancers (27), myopathies (28), prior anterior cervical fusion (29), and sarcopenia (30). This condition precipitates a cascade of severe complications: insufficient intake leads to malnutrition (30), electrolyte imbalances, dehydration, and disruptions of the internal milieu, which in turn compromise immune function. Furthermore, aspiration-induced pneumonia (23) remains a critical threat. Collectively, these complications are closely linked to a poor prognosis. Previous studies have highlighted the high mortality in this population; for instance, Lima et al. (31) reported a 30-day mortality rate of 13% in patients with severe dysphagia undergoing PEG. In our study, despite the strict exclusion of patients with terminal malignancies or those undergoing PEG solely for gastric decompression, and the provision of nutritional support via PEG or TPN, the mortality burden remained substantial. We observed 63 deaths (25.4%) within 180 days and 100 deaths (40.3%) within 360 days, with a median survival time of 474 days (95% CI: 362–699 days). Given this limited survival window, the early identification of high-risk patients using biomarkers is of critical clinical importance.

The prognostic association between LCR and outcomes observed in our study appears biologically plausible, as this composite index may reflect the balance between host immune competence, nutritional reserve, and systemic inflammatory burden. These findings align with those of Masaki et al. (32), who evaluated prognostic factors in elderly patients with dysphagia undergoing PEG. They demonstrated that abdominal wall thickness (AWT) was significantly associated with survival, identifying TLC as a key predictor of AWT. Furthermore, regarding inflammatory markers, they reported significantly lower CRP levels in the high-AWT group compared to the low-AWT group (0.55 vs. 1.06 mg/dl, *P* = 0.003), emphasizing the critical roles of both TLC and CRP. Our results corroborate the value of these components. In patients with severe dysphagia, a low LCR characterized by lymphopenia and elevated CRP may reflect a poor prognosis, consistent with Masaki's observations on immune-nutritional depletion. Mechanistically, this phenomenon likely reflects a vicious cycle between impaired host defense and systemic inflammation. While lymphopenia compromises the regulation of immune responses and pathogen clearance, the concurrent systemic inflammation reflected by elevated CRP suppresses lymphopoiesis and accelerates lymphocyte apoptosis. Ultimately, this cycle accelerates the depletion of physiological reserves, rendering patients highly vulnerable to the dual burden of severe infections and hypercatabolic wasting.

Although prospective studies specifically evaluating LCR in geriatric patients with dysphagia remain limited, previous prospective or longitudinal cohort studies in other clinical settings have investigated LCR as a prognostic marker reflecting inflammatory burden, nutritional impairment, and immune dysfunction. Previous studies have reported the prognostic value of LCR in malignancies, including colorectal (33), gastric (21), esophageal (16), and hepatocellular carcinomas (34, 35), as well as in hemodialysis (36), sepsis (17), myocardial infarction (20), heart failure (19), and fractures (18, 37). These studies collectively suggest that LCR may function as an integrated biomarker linking systemic inflammation, immune reserve, nutritional impairment, and clinical outcomes. For example, in patients with heart failure, a lower log-transformed LCR was associated with worse nutritional indices and poorer physical function. Furthermore, regarding 2-year survival, a higher log-transformed LCR was indicated to predict a better prognosis (HR: 0.88; 95% CI: 0.81–0.95; *P* = 0.002) (19). Similarly, in a cohort of hemodialysis patients followed for a median of 75.1 months, the optimal LCR cut-off was identified as 1,513.1; patients with an LCR ≥ 1,513.1 exhibited significantly reduced mortality risk (HR: 0.75, 95% CI: 0.66–0.85, *P* < 0.001) (36). In acute settings such as sepsis (*n* = 1,123), multivariate Cox analyses confirmed LCR as a useful predictor of 30-day mortality, with each unit increase in LCR significantly lowering the risk of death (HR: 0.370; 95% CI: 0.142–0.963; *P* = 0.042) (17). Our secondary analysis showed a similar association in geriatric patients with severe dysphagia requiring artificial nutrition [adjusted HR: 0.89; 95% CI: 0.83–0.97 for log_2_(LCR)]. These findings extend the potential clinical applicability of LCR to a frail dysphagic population in whom inflammation, malnutrition, impaired immunity, aspiration risk, and functional decline frequently coexist.

However, it is instructive to compare these findings with research in other critical conditions. In sepsis patients, LCR appeared to show improved prognostic accuracy for 30-day mortality compared with traditional indices like white blood cell count, lymphocyte count, CRP, and albumin (17). A cohort of hemodialysis patients also showed that LCR's predictive value tended to decline over extended periods, with AUCs dropping from 0.620 at 1 year to 0.549 at 7 years (36). Our time-dependent ROC analyses suggested strong predictive ability within the first year, with AUCs ranging from 0.760 (95% CI: 0.688–0.833) at 90 days to 0.713 (0.645–0.782) at 360 days. This predictive capacity, however, attenuated over extended follow-up, dropping to 0.623 (0.446–0.801) by 1,080 days. Previous studies based on this specific cohort have confirmed the value of albumin-based indicators, such as the Prognostic Nutritional Index and the C-reactive protein/albumin ratio (15, 38, 39), and other research has identified anemia as a predictor of 4-week mortality in patients with dysphagia (31). However, these studies did not examine time-dependent changes in predictive ability. Given LCR's role as an inflammatory and immune marker, its value at admission may be pre-dominantly predictive of short-term mortality risk. As the acute phase resolves, the impact of these baseline markers on survival could diminish. Our study indicated that the AUC gradually declined to below 0.7 over time. Time-dependent ROC and Schoenfeld residual analyses reveal that the prognostic value of log_2_(LCR) is significant in the first year post-admission; thereafter, its predictive efficacy gradually declines. In our study, the predictive performance of LCR for 1-year mortality was evaluated against its individual components (CRP and TLC) and established immunonutritional indices (albumin, CAR, and PNI). Incorporating log_2_(LCR) into the baseline model appeared to offer improvements beyond those of CRP- and CAR-based models. DeLong testing indicated comparable predictive performance among log_2_(LCR), TLC, PNI, and albumin. Although previous research has explored the utility of LCR across various clinical settings (18, 22, 33, 40), further evaluation of its performance against other immune-nutritional indices remains necessary. A recent study of hospitalized older adults identified both LCR and PNI as predictors of in-hospital mortality (40), yet direct statistical comparisons between these markers remain scarce. In our study, applying the Boruta machine-learning algorithm for advanced feature selection revealed that log_2_(LCR) carried the highest predictive weight among all evaluated immune-nutritional markers. The current study broadens these findings by comprehensively comparing log_2_(LCR) against its individual components and competing biomarkers. Clinically, this suggests that log_2_(LCR) has the potential to serve as a practical stratification tool for assessing 1-year mortality in geriatric patients with severe dysphagia requiring artificial nutrition.

To further validate the robustness of this marker's predictive ability for 1-year mortality, we employed PSM, IPTW, PA, and other sensitivity analyses. These results consistently suggested that the high log_2_(LCR) group tended to have a survival advantage within the first year, reinforcing the idea that log_2_(LCR) may serve as a useful stratification marker for assessing 1-year mortality. For those surviving beyond 1 year, long-term outcomes appear to be more closely associated with dynamic factors such as care quality, recurrence of aspiration, and management of chronic comorbidities, while the influence of the baseline immune-nutritional status at admission gradually diminishes.

From a clinical perspective, LCR may serve as a practical tool for prognostic assessment of 1-year mortality in geriatric patients with severe dysphagia receiving artificial nutrition. Incorporating early LCR assessment into dysphagia management may help identify high-risk patients and enable timely intervention. A low LCR may indicate impaired immune-nutritional status and greater systemic inflammation, suggesting the need for closer monitoring, more intensive nutritional assessment, infection surveillance, fluid and electrolyte management, and respiratory care. Given the high susceptibility of these patients to malnutrition, fluid and electrolyte disturbances, and aspiration pneumonia, timely multidisciplinary management is essential to improve outcomes (41, 42). Notably, because LCR is derived from routinely available CRP and lymphocyte count, it does not require additional cost or specialized testing. These findings support the use of LCR for prognostic stratification and may facilitate its broader adoption in clinical practice.

This study has several limitations. First, its retrospective secondary analysis design, coupled with a specific cohort of elderly patients with severe dysphagia requiring artificial nutrition, may limit the generalizability of our findings, particularly to patients with mild to moderate dysphagia who tolerate oral feeding. Second, despite controlling for various confounders, the influence of unmeasured or unknown factors cannot be entirely ruled out. Third, given the temporal variations in LCR's prognostic ability, a single measurement at admission may not fully capture long-term dynamic risks. Finally, although the use of publicly available de-identified data supports transparency and reproducibility, future prospective multicenter longitudinal studies are needed to externally validate our findings and evaluate whether monitoring temporal changes in LCR can further improve prognostic assessment.

## Conclusion

In geriatric patients with severe dysphagia requiring artificial nutrition, LCR shows an inverse association with mortality. While its predictive value may weaken over extended follow-up, LCR remains a robust indicator for 1-year mortality. Early LCR assessment could help identify high-risk patients, potentially guiding prompt interventions to improve outcomes.

## Data Availability

Publicly available datasets were analyzed in this study. This data can be found here: Dryad Digital Repository (doi: 10.5061/dryad.gg407h1): https://datadryad.org/dataset/doi:10.5061/dryad.gg407h1.
