# Supplementary material for: Prognostic value of the lymphocyte-to-C-reactive protein ratio for mortality in geriatric patients with severe dysphagia requiring artificial nutrition: a retrospective secondary analysis
Source: Front Med (Lausanne). 2026 Jun 9;13:1823539. doi: 10.3389/fmed.2026.1823539 (PMC13286769; doi:10.3389/fmed.2026.1823539)
Supplement: Supplementary file 1 [file Data_Sheet_1.docx]

**Supplementary material**

**Supplementary Table 1.** Results of univariate Cox proportional hazards analysis for mortality;

**Supplementary Table 2.** Covariate selection: effect size change exceeding 10% and VIF < 5;

**Supplementary Table 3** Feature importance and selection for 1-year mortality using Boruta Algorithm;

**Supplementary Table 4.** Multivariable Cox regression of log₂(LCR) and mortality in the non-imputed dataset;

**Supplementary Figure 1.** Flowchart of patient selection;

**Supplementary Figure 2.** Restricted cubic spline curves for mortality by log₂(LCR);

**Supplementary Figure 3.** Kaplan-Meier survival curve stratified by the optimal log₂(LCR) cutoff;

**Supplementary Figure 4.** Forest plot of sensitivity analyses comparing high (≥ 11.67) vs. low (< 11.67) log₂(LCR).

**Supplementary Table 1.** Results of univariate Cox proportional hazards analysis for mortality.

| Variable | HR (95% CI) | P value |
| --- | --- | --- |
| Log₂(LCR) (continuous) | 0.82 (0.76, 0.87) | < 0.001 |
| PEG (Yes vs. No) | 0.28 (0.20, 0.40) | < 0.001 |
| Age (years) | 1.05 (1.02, 1.07) | < 0.001 |
| Sex (Female vs. Male) | 0.45 (0.32, 0.63) | < 0.001 |
| Body mass index (kg/m²) | 0.98 (0.93, 1.03) | 0.512 |
| Serum albumin (g/dL) | 0.37 (0.28, 0.50) | < 0.001 |
| Hemoglobin (g/dL) | 0.77 (0.71, 0.83) | < 0.001 |
| Oral intake, (Yes vs. No) | 0 (0, Inf) | 0.994 |
| Comorbidities (Yes vs. No) |  |  |
| Cerebrovascular disease | 0.55 (0.39, 0.78) | < 0.001 |
| Dementia | 1.95 (1.39, 2.75) | < 0.001 |
| Neuromuscular disease | 0.82 (0.38, 1.76) | 0.610 |
| Aspiration pneumonia | 1.76 (1.25, 2.48) | 0.001 |
| Ischemic heart disease | 1.97 (1.32, 2.93) | < 0.001 |
| Congestive heart failure | 2.02 (1.44, 2.84) | < 0.001 |
| Chronic lung disease | 1.43 (0.79, 2.59) | 0.237 |
| Chronic liver disease | 1.39 (0.73, 2.65) | 0.314 |
| Chronic kidney disease | 2.59 (1.79, 3.74) | < 0.001 |
| Sepsis | 2.35 (1.53, 3.62) | < 0.001 |

**Abbreviations:** CI, confidence interval; HR, hazard ratio; LCR, lymphocyte-to-C-reactive protein ratio; PEG, percutaneous endoscopic gastrostomy.**Supplementary Table 2.** Covariate selection: effect size change exceeding 10% and VIF < 5.

| **Variable** | **Change in Estimate I (%)** | **Change in Estimate II (%)** | **VIF** | **Select** |
| --- | --- | --- | --- | --- |
| Crude | Ref. | Ref. | 1.26 | Ref. |
| PEG | -9.5 | -14.5 | 1.51 | Yes |
| Age (years) | -10.1 | -9.7 | 1.28 | Yes |
| Sex (Female vs Male) | -1.9 | 6.5 | 1.59 | No |
| Cerebrovascular disease | -7.1 | 4.0 | 1.76 | No |
| Dementia | -3.8 | 0.5 | 1.70 | No |
| Neuromuscular disease | 1.5 | -0.4 | 1.32 | No |
| Aspiration pneumonia | -6.8 | -3.2 | 1.57 | No |
| Ischemic heart disease | -8.3 | 4.2 | 1.60 | No |
| Congestive heart failure | -9.5 | 4.7 | 1.57 | No |
| Chronic lung disease | 0.3 | -0.2 | 1.19 | No |
| Chronic liver disease | 1.1 | -6.8 | 1.20 | No |
| Chronic kidney disease | -7.3 | -1.9 | 2.06 | No |
| Serum albumin (g/dL) | -41.8 | 8.7 | 1.52 | Yes |
| Hemoglobin (g/dL) | -31.5 | 8.6 | 1.76 | Yes |
| Body mass index (kg/m²) | -0.4 | -1.4 | 1.18 | No |
| Oral intake | -3.4 | 1.0 | 1.00 | No |
| Sepsis | -0.9 | 4.1 | 1.23 | No |

Abbreviations: PEG, percutaneous endoscopic gastrostomy; CRP, C-reactive protein; LCR, lymphocyte-to-CRP ratio. Log₂(LCR) denotes the base-2 logarithm of LCR. VIF, variance inflation factor;

**Supplementary Table 3** Feature importance and selection for 1-year mortality using Boruta Algorithm

| Feature | Mean Importance | Median Importance | Min Importance | Max Importance | Normalised Hits | Decision |
| --- | --- | --- | --- | --- | --- | --- |
| age | 6.05 | 6.07 | 2.36 | 8.89 | 0.97 | Confirmed |
| sex | 5.51 | 5.58 | 2.85 | 7.88 | 0.94 | Confirmed |
| CI | 0.35 | 0.46 | -1.44 | 1.76 | 0.00 | Rejected |
| dement | 2.26 | 2.30 | -1.15 | 5.16 | 0.38 | Tentative |
| IHD | -0.18 | -0.47 | -1.93 | 1.38 | 0.00 | Rejected |
| CHF | 3.63 | 3.74 | 0.88 | 5.50 | 0.72 | Confirmed |
| CKD | 3.99 | 3.98 | 1.05 | 6.10 | 0.81 | Confirmed |
| asp | 0.94 | 0.95 | -0.22 | 2.29 | 0.00 | Rejected |
| sepsis | 2.54 | 2.56 | -0.17 | 4.74 | 0.53 | Tentative |
| Alb | 4.76 | 4.77 | 2.76 | 6.74 | 0.92 | Confirmed |
| Hb | 6.60 | 6.65 | 4.52 | 8.84 | 0.98 | Confirmed |
| log2(LCR) | 12.83 | 12.74 | 10.31 | 14.63 | 1.00 | Confirmed |
| TLC | 11.13 | 11.23 | 7.77 | 13.58 | 1.00 | Confirmed |
| CRP | 7.12 | 7.05 | 4.70 | 9.84 | 0.98 | Confirmed |
| CAR | 7.86 | 7.84 | 5.81 | 10.63 | 0.99 | Confirmed |
| PNI | 10.03 | 10.12 | 7.61 | 12.41 | 1.00 | Confirmed |
| PEG | 13.16 | 13.17 | 10.82 | 16.06 | 1.00 | Confirmed |

**Abbreviations:** asp: Aspiration pneumonia; CAR: C-reactive protein-to-albumin ratio; CHF: congestive heart failure; CI: Cerebrovascular disease; CKD: chronic kidney disease; CRP: C-reactive protein; dement: dementia; IHD: ischemic heart disease; LCR: lymphocyte-to-C-reactive protein ratio; PEG: percutaneous endoscopic gastrostomy; PNI: prognostic nutritional index; TLC: total lymphocyte count.

**Supplementary Table 4.** Multivariable Cox regression of log₂(LCR) and mortality in the non-imputed dataset

| Variable | No. Events (%) | Model 1 | | Model 2 | | Model 3 | | Model 4 | |
| --- | --- | --- | --- | --- | --- | --- | --- | --- | --- |
|  |  | HR (95% CI) | P **Value** | HR (95% CI) | P **Value** | HR (95% CI) | P **Value** | HR (95% CI) | P **Value** |
| Log₂(LCR) | 132 (53.9) | 0.82 (0.76-0.88) | <0.001 | 0.84 (0.78-0.90) | <0.001 | 0.86 (0.80-0.92) | <0.001 | 0.90 (0.83-0.97) | 0.006 |
| Tertile of log₂(LCR) |  |  |  |  |  |  |  |  |  |
| T1 (< 9.02) | 56 (69.1) | 1.00 (Ref.) |  | 1.00 (Ref.) |  | 1.00 (Ref.) |  | 1.00 (Ref.) |  |
| T2 (9.03-11.68) | 52 (63.4) | 0.70 (0.48-1.02) | 0.066 | 0.75 (0.51-1.10) | 0.139 | 0.79 (0.54-1.16) | 0.233 | 0.79 (0.52-1.19) | 0.258 |
| T3 (> 11.69) | 24 (29.3) | 0.26 (0.16-0.42) | <0.001 | 0.31 (0.19-0.50) | <0.001 | 0.34 (0.21-0.56) | <0.001 | 0.45 (0.27-0.77) | 0.003 |
| P for trend |  |  | <0.001 |  | <0.001 |  | <0.001 |  | 0.003 |

Model 1: Unadjusted.

Model 2: Adjusted for sex and age.

Model 3: Adjusted for variables in Model 2 plus cerebrovascular disease, dementia, chronic kidney disease, congestive heart failure, and ischemic heart disease.

Model 4: Adjusted for variables in Model 3 plus percutaneous endoscopic gastrostomy, aspiration pneumonia, sepsis, hemoglobin, and serum albumin.

**Abbreviations:** HR, hazard ratio; CI, confidence interval; LCR, lymphocyte-to-CRP ratio; CRP, C-reactive protein.


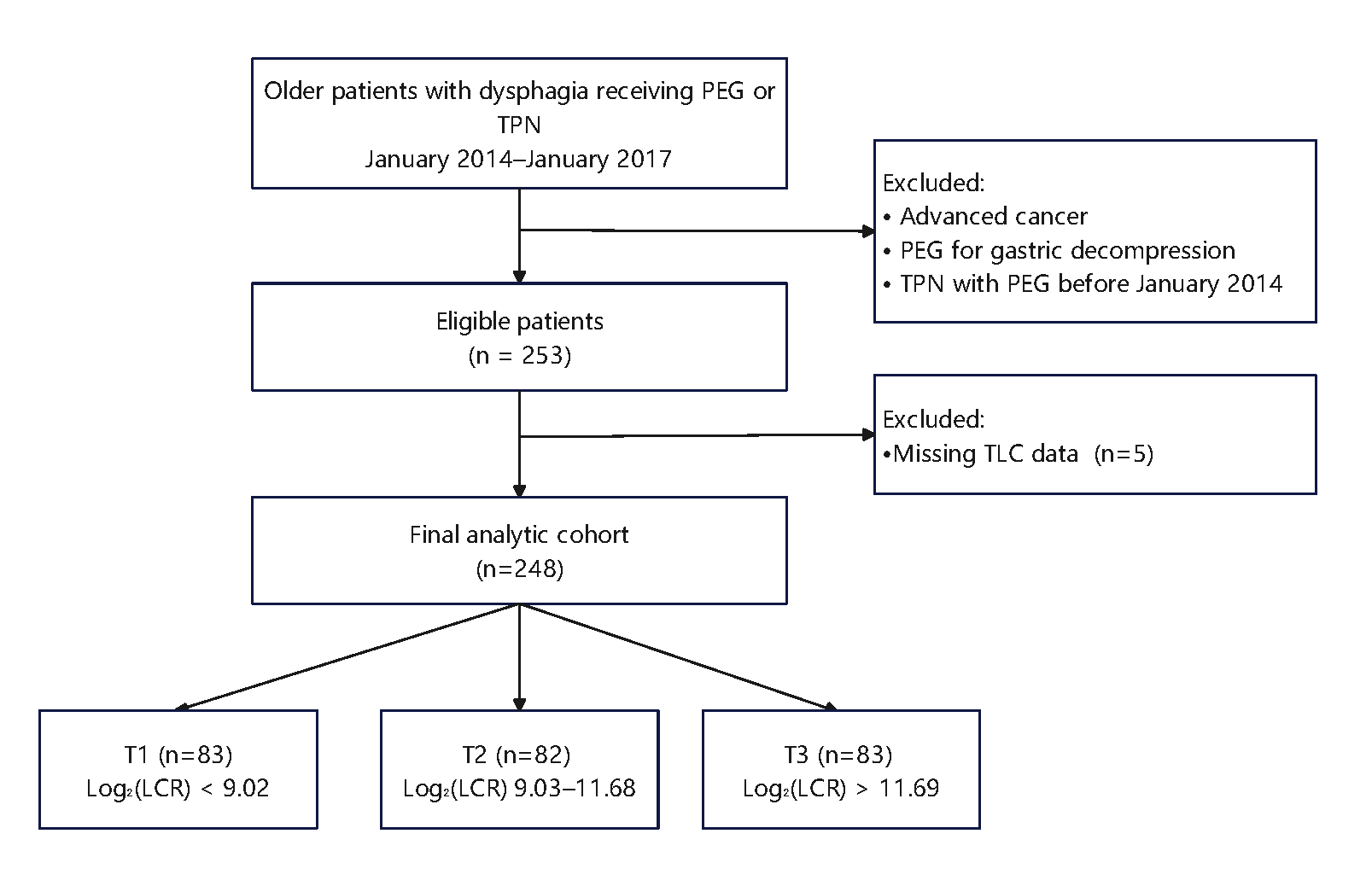


**Supplementary Figure 1. Flowchart of patient selection. Abbreviations:** PEG, percutaneous endoscopic gastrostomy; TPN, total parenteral nutrition; TLC, total lymphocyte count; LCR, lymphocyte-to-C-reactive protein ratio.


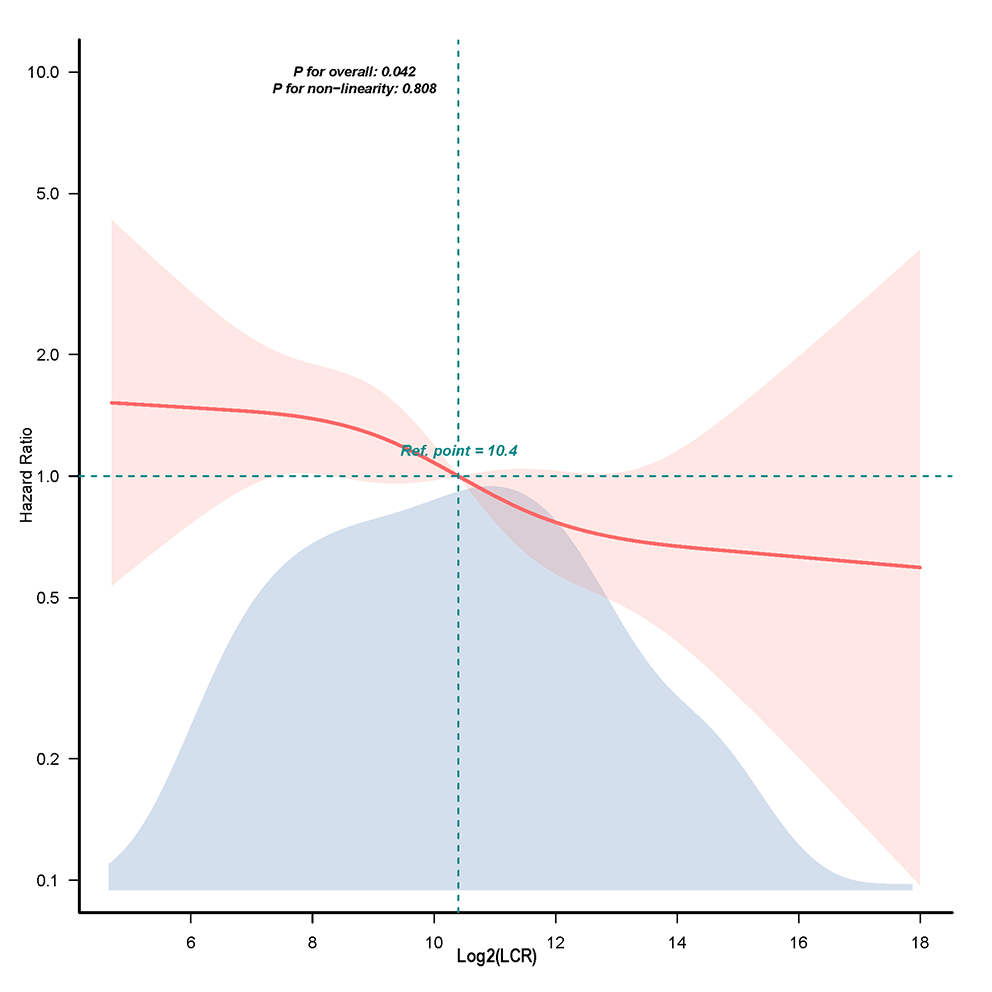


**Supplementary Figure 2. Restricted cubic spline curves for mortality by log₂(LCR).** The heavy central line represents the estimated adjusted hazard ratio, with the shaded band denoting the 95% confidence interval. The model was adjusted for age, sex, cerebrovascular disease, severe dementia, chronic kidney disease, congestive heart failure, ischemic heart disease, percutaneous endoscopic gastrostomy, aspiration pneumonia, sepsis, hemoglobin, and serum albumin. Abbreviation: LCR, lymphocyte-to-C-reactive protein ratio.


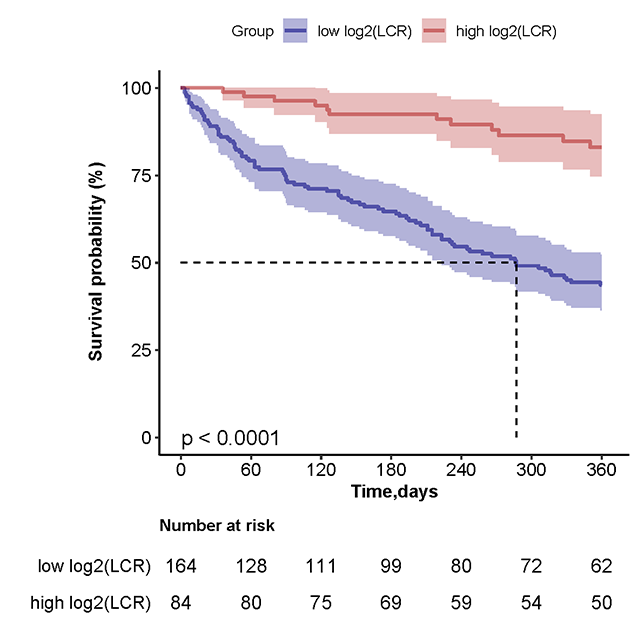


**Supplementary Figure 3. Kaplan-Meier survival curve stratified by the optimal log₂(LCR) cutoff.** Patients were divided into high (≥11.67) and low (<11.67) log2(LCR) groups based on the optimal cutoff determined by maximally selected rank statistics. The analysis was restricted to the first 360 days of follow-up. **Abbreviation**: LCR, lymphocyte-to-C-reactive protein ratio.


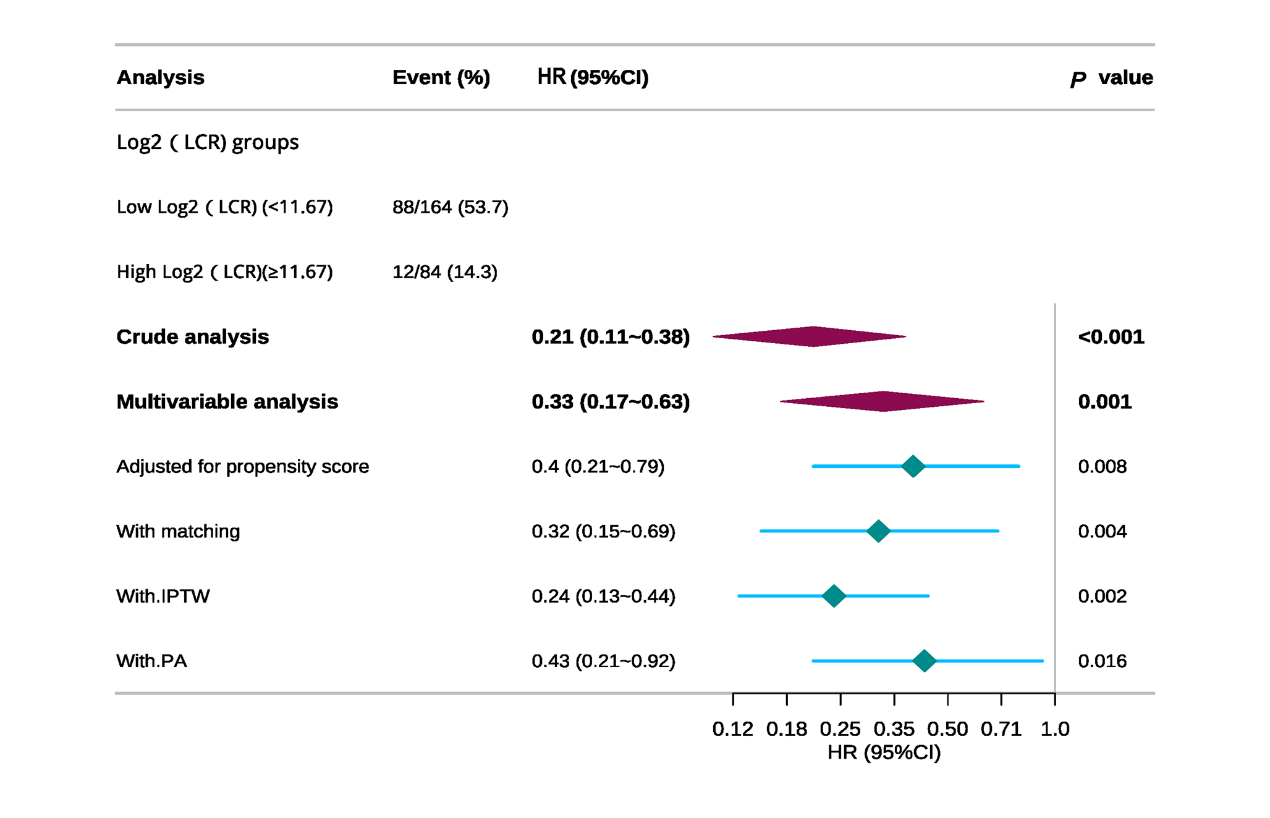


**Supplementary Figure 4. Forest plot of sensitivity analyses comparing high (≥ 11.67) vs. low (< 11.67) log₂(LCR).** Models include (1) Crude; (2) Multivariable; (3) Propensity score adjustment; (4) Propensity score matching (PSM); (5) Inverse probability of treatment weighting (IPTW); and (6) Pairwise algorithm (PA). **Abbreviation:** LCR, lymphocyte-to-C-reactive protein ratio.
